# Exploration of Virtual Candidates for Human HMG-CoA Reductase Inhibitors Using Pharmacophore Modeling and Molecular Dynamics Simulations

**DOI:** 10.1371/journal.pone.0083496

**Published:** 2013-12-30

**Authors:** Minky Son, Ayoung Baek, Sugunadevi Sakkiah, Chanin Park, Shalini John, Keun Woo Lee

**Affiliations:** 1 Division of Applied Life Science (BK21 Plus Program), Systems and Synthetic Agrobiotech Center (SSAC), Plant Molecular Biology and Biotechnology Research Center (PMBBRC), Research Institute of Natural Science (RINS), Gyeongsang National University (GNU), Gazwa-dong, Jinju, Republic of Korea; 2 Department of Chemistry and Biochemistry, University of California Los Angeles, Los Angeles, California, United States of America; University of Edinburgh, United Kingdom

## Abstract

3-hydroxy-3-methylglutaryl coenzyme A reductase (HMGR) is a rate-controlling enzyme in the mevalonate pathway which involved in biosynthesis of cholesterol and other isoprenoids. This enzyme catalyzes the conversion of HMG-CoA to mevalonate and is regarded as a drug target to treat hypercholesterolemia. In this study, ten qualitative pharmacophore models were generated based on chemical features in active inhibitors of HMGR. The generated models were validated using a test set. In a validation process, the best hypothesis was selected based on the statistical parameters and used for virtual screening of chemical databases to find novel lead candidates. The screened compounds were sorted by applying drug-like properties. The compounds that satisfied all drug-like properties were used for molecular docking study to identify their binding conformations at active site of HMGR. The final hit compounds were selected based on docking score and binding orientation. The HMGR structures in complex with the hit compounds were subjected to 10 ns molecular dynamics simulations to refine the binding orientation as well as to check the stability of the hits. After simulation, binding modes including hydrogen bonding patterns and molecular interactions with the active site residues were analyzed. In conclusion, four hit compounds with new structural scaffold were suggested as novel and potent HMGR inhibitors.

## Introduction

**Figure 1:**
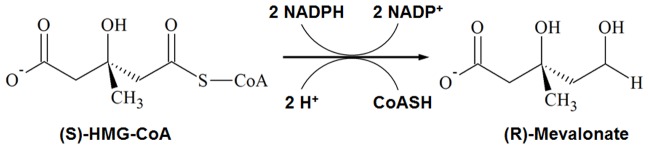
A biochemical reaction catalyzed by HMGR. HMG-CoA is converted to mevalonte using two molecules of NADPH. The reaction proceeds by two successive hydride transfers.

**Figure 2:**
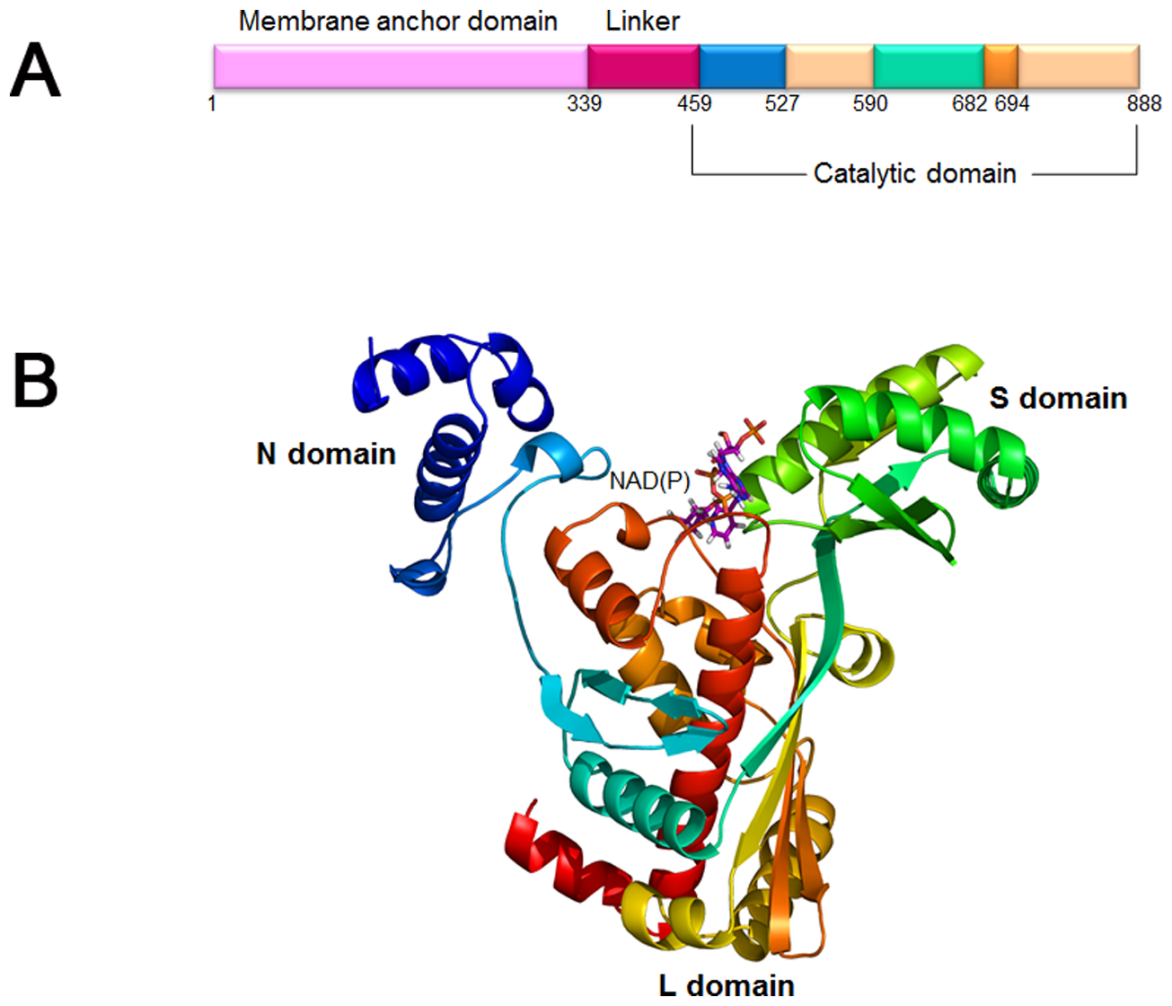
Functional domain and 3D structures of the *human* HMGR. (A) *Human* HMGR consists of 888 amino acids and is divided into three domains: membrane anchor domain, linker domain, and catalytic domain. In the catalytic domain, further separated into three subdomain named as N domain, L domain, and S domain. Cis-loop which acts as HMG binding pocket is present between S and L domain. (B) Crystal structure of the *human* HMGR monomer with NAD (P), cofactor (PDB ID: 1DQA). The protein was represented as cartoon model and this figure was prepared using PyMOL.

3-hydroxy-3-methylglutaryl coenzyme A reductase (HMGR) is a rate-controlling enzyme in the mevalonate pathway which is mainly involved in biosynthesis of cholesterol and other isoprenoids [1]. This enzyme catalyzes the four-electron reduction of HMG-CoA to coenzyme A and mevalonate which is the precursor of isoprenoids (Figure 1) [2]–[4]. Two molecules of NADPH are used and the reaction progresses by two successive hydride transfers [3]. Since this reaction is the first committed step in cholesterol biosynthesis in mammals, HMGR is considered as a primary target enzyme to treat hypercholesterolemia [5]. *Human* HMGR comprises of a single polypeptide chain of 888 amino acids and is divided into three domains: membrane anchor domain, linker domain, and catalytic domain (Figure 2A). The membrane anchor domain (residues 1–339) of the protein locates in the endoplasmic reticulum membrane and catalytic domain (residues 460–888) of the protein is present in the cytoplasm. A linker region (residues 340–459) connects the membrane anchor domain and the catalytic domain of the protein [2]. Structurally, the catalytic domain of HMGR is further separated into three sub-domains namely N domain, L domain, and S domain (Figure 2B). The L domain is a large central domain which has a dimerization motif ENVIG. The N and S domains are small helical domains, in particular, the S domain includes an NAD(P) binding motif DAMGMN and is inserted into the L domain [2]. The binding pocket for HMG-CoA is characterized by a cis-loop (residues 682–694). The crystal structures of *human* HMGR show the catalytic portions of the enzyme form a tetramer and each active site is located at the interface of two monomers. The homodimer is a functional unit of the enzyme. Moreover, the formation of the tetramer does not seem to affect the substrate binding [3].

High cholesterol levels have been identified as a primary risk factor of coronary artery disease. Even though advances in diagnosis and treatment exist, this disease is still sometimes fatal as well as a major problem in developed countries, responsible for about 36% of deaths in 2004 in the United States [6]. As shown in large-scale clinical trials, inhibition of HMGR significantly reduces the cholesterol levels and decreases the risks of stroke by 29% and overall mortality by 22% [7]. Inhibitors of HMGR are commonly referred to as statins, and are very efficient in lowering serum cholesterol levels. Until recently, about seven statins which disrupt the rate limiting step of cholesterol synthesis are available or in late-stage clinical development [4], [8]. All statins have conserved HMG-like moiety and inhibit HMGR by occupying the active site of the enzyme in place of the substrate. Previously, several studies reported that statins competitively inhibit HMG-CoA but do not involve in NADPH binding [2], [4], [9]–[11]. Although statins are generally known as the most effective anti-hypercholesterolemia drug, they have various adverse effects including distal muscle weakness, headache, increases in serum levels of hepatic transaminases, and sleep disorders in controlled tests. Moreover, side effects such as eczema, peripheral neuropathy, sensory disturbances, and depression have been shown on prolonged use of the statins [12]–[15].

For this reason, we aimed to discover novel scaffolds of HMGR inhibitor using pharmacophore modeling and molecular dynamics (MD) simulation approaches. Pharmacophore-based virtual screening is one of the most effective and useful tools in the field of drug discovery to save time and cost [16]–[21]. In this study, we generated ligand based pharmacophore models and followed this by virtual screening of various databases. Subsequently molecular docking studies were performed to find suitable binding orientations for new drug-like compounds. Finally, the selected hit compounds were subjected to MD simulations to further evaluate the stability of their binding in the active site of HMGR.

## Materials and Methods

### Training and test sets preparation

We have collected 139 HMGR inhibitors from the literature [22]–[37] and binding databases [38], [39] to generate and validate the pharmacophore hypotheses. These compounds have HMGR inhibitory activities, expressed in IC_50_ values. Of these, 7 compounds were selected as a training set and the remaining compounds were defined as a test set. These compounds were minimized to a local energy minimum using the CHARMm force field implemented within Discovery Studio (DS) 3.1. This generates a conformational model for each compound in the training set that represents its flexibility and also promotes a conformational variation that forces similar conformer away from each other. DS provides two types of conformational analysis using the poling algorithm: *FAST* and *BEST*. Both methods search for a set of representative conformers for each compound and automatically build as many conformers as required for adequate coverage, up to a user-specifiable maximum. The *FAST* conformational analysis method is mostly used for a database generation because the tolerances can be adjusted to minimize the effect of incomplete conformational coverage. The *BEST* conformational analysis is a method of choice if the conformational models are to be used for hypothesis generation. The conformational model will be used not only in hypothesis generation but also for fitting the compounds to a hypothesis. In this study, the *BEST* conformational analysis was applied to all compounds in the training and test sets.

### Pharmacophore model generation and validation

We have performed pharmacophore model generation using the *Hip-Hop* algorithm which attempts to find chemical features shared by the HMGR inhibitors in the training set. The common features hypotheses were developed based on pharmacophoric features such as hydrogen bond acceptor (HBA), hydrogen bond donor (HBD), ring aromatic (RA), and hydrophobic (HYP) groups using common feature generation module in DS. The *Minimum Interfeature Distance* was set to 1.5 Å. The parameters, *Number of Leads That May Miss* and *Feature Misses*, were used as a value of ‘1’ to allow that one of the compounds may not contain all the features when building hypothesis space. The *Complete Misses* option, which is used for specifying the number of compounds that do not have to map to any features in the hypothesis, was set to ‘0’. As we defined the number of maximum pharmacophores as 10, the generation run returns 10 possible hypotheses having different arrangement of constituent features and sorts them according to ranking scores. To select the best pharmacophore model among the generated hypotheses, test set compounds containing known 132 inhibitors were screened using *Best Flexible Search* option in DS.

### Virtual screening

Virtual screening of chemical databases is an effective and rapid method to obtain new compounds with desired activity profiles. The screening process was performed to find a novel and potent compound for further drug development. The best hypothesis was used in the screening of various databases, Chembridge, Maybridge, NCI, and Asinex. The compounds which satisfy the chemical features present in the best hypothesis were chosen and sorted by applying drug-like properties like Lipinski's rule of five [40] and ADMET (Absorption, Distribution, Metabolism, Excretion and Toxicity) [41]. According to the rule of five, compounds are considered likely to be well absorbed when they possess LogP less than 5, molecular weight less than 500, the number of hydrogen bond donors less than 5, the number of hydrogen bond acceptors less than 10, and the number of rotatable bonds less than 10. The ADMET properties were used to estimate values of blood brain barrier (BBB) penetration, solubility, cytochrome P450 2D6 (CYP2D6) inhibition, hepatotoxicity, human intestinal adsorption (HIA), plasma protein binding (PPB), and to assess a broad range of toxicity measure of the compounds. Among all these criteria we mainly focused on BBB penetration, solubility, and adsorption and the cut off value was 3, 3, and 0, respectively. All these properties were calculated using DS. The resultant hit compounds were used in molecular docking studies to find whether these compounds can form suitable binding orientations in the active site of HMGR.

### Molecular docking calculations

The training set and the screened compounds were subjected to molecular docking calculation using GOLD 5.0.1 (Genetic Optimization for Ligand Docking) [42], [43]. It is an automated docking program that uses a genetic algorithm to explore the ligand conformation. The three dimensional crystal structure of HMGR in complex with HMG, CoA, and NADP^+^ (PDB ID: 1DQA) was taken from Protein Data Bank (PDB, www.rcsb.org) and the dimeric structure was prepared to perform the docking calculations and MD simulation. All water molecules were removed and hydrogen atoms were added. The protein residues within the radius of 8 Å around the center of mass of the HMG substrate were chosen to define an interaction sphere during the docking calculation. Maximum saved conformation was set to 10. The generated conformations were ranked according to their GOLD fitness score. Default cut off values of 2.5 Å for hydrogen bond and 4.0 Å for the van der Waals distance were applied. Final hit compounds were selected based on the fitness score and molecular interactions with critical residues of HMGR.

### Molecular dynamics simulations

MD simulations for the HMGR in complex with rosuvastatin, atorvastatin, and the final hit compounds were performed using GROMACS 4.5.3 package [44], [45] with AMBER force field to gain a better relaxation and a correct arrangement of atoms as well as to refine side chain orientation of HMGR. Topology files for the ligands were generated using ACPYPE (AnteChamber PYthon Parser interface) [46]. Ionization states for all ligands were set corresponding to a neutral pH. The structure was solvated in a cubic box and the TIP3P water model [47], [48] was used to create an aqueous environment. The system was neutralized by replacing water molecules with Na^+^ counter ions. It was subjected to a steepest descent energy minimization until a tolerance of 1000 kJ/mol, to avoid high energy interactions and steric clashes. The energy minimized system was treated for 100 ps in an equilibration run. The pre-equilibrated system was consequently subjected to a 10 ns production run with a time-step of 2 fs. A constant temperature and pressure of 300 K and 1 bar were maintained during the simulations with the V-rescale thermostat [49] and the Parrinello-Rahman barostat [50]. All bond lengths were constrained with the LINCS [51] method. The particles mesh Ewald (PME) electrostatic [52], [53] and periodic boundary conditions were applied in all directions. The cut off value of 9 Å cut off for coulomb interactions and 10 Å for van der Waals interactions were assigned. From the trajectories during last 2 ns of the MD simulations, a frame which showed the closest to an average structure was selected as a representative structure. Further analyses were done using the representative structures. For a validation purpose, we also calculated binding free energies for the final hit compounds using AutoDock 4.2 [54]–[56]. It predicts interactions between ligands and biomolecules and also estimates the binding free energy using Lamarckian Genetic Algorithm. Three-dimensional grid was generated based on the docked conformation of the compound via AutoGrid implemented in the AutoDock.

## Results and Discussion

### Generation and validation of common feature pharmacophore models

**Figure 3:**
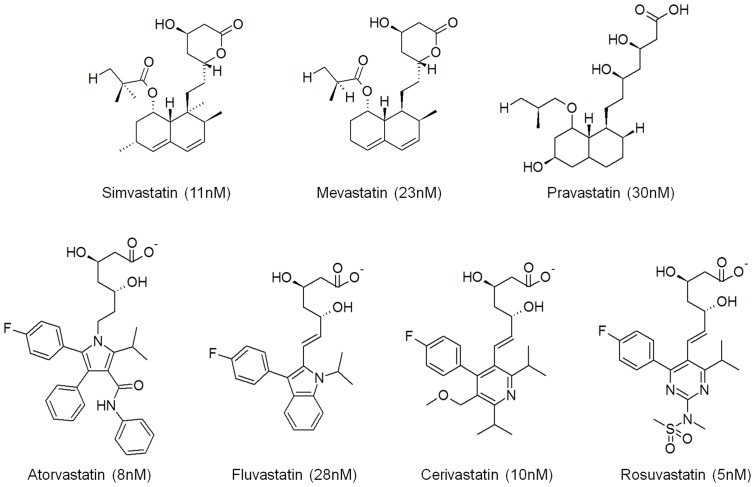
The chemical structures of *human* HMGR inhibitors used in pharmacophore model generation along with their IC_50_ values.

**Figure 4:**
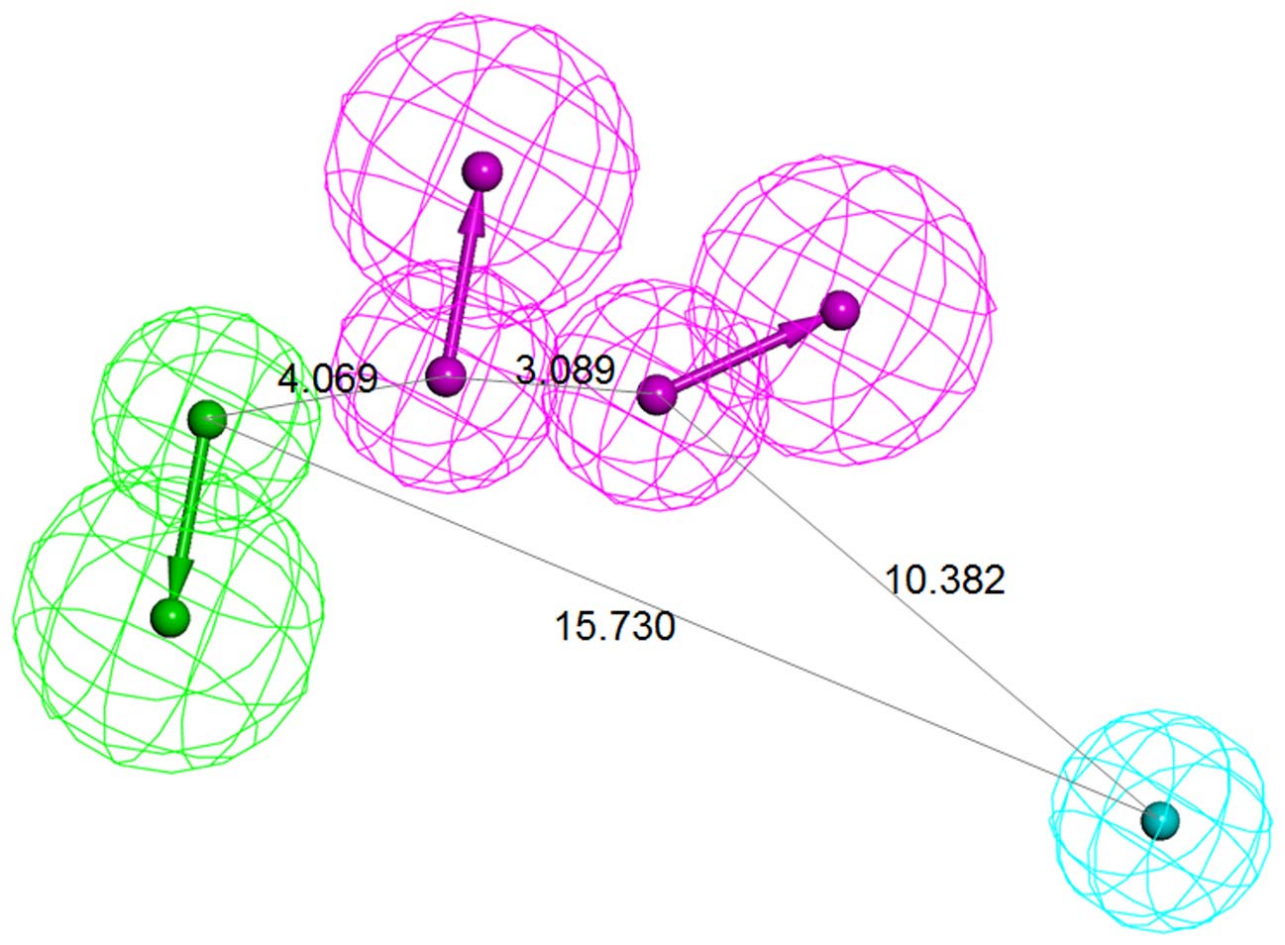
The generated pharmacophore model with their inter-feature distance constraints. HBA, HBD, and HYP features are displayed in green, magenta, and cyan, respectively.

7 Highly active HMGR inhibitors were chosen as the training set for the generation of common feature pharmacophore models. The molecular structures and IC_50_ values of the training set are shown in Figure 3. The training set compounds should cover adequate structural diversities to elucidate the production of the pharmacophore models which are responsible for inhibiting the activity of the particular targets. The different functional groups in the inhibitors may adopt different binding modes to maximize their interaction in the active site of the protein. In the *Common feature generation* module implemented in DS, the compounds that are used to construct the pharmacophore configuration space can be specified through setting the parameters including principal value. Table 1 reports the top 10 pharmacophore models generated using the training set. Hypo1 to 3 consisting of one HYP, one HBD, and two HBA features have a ranking score of 81.88. In case of Hypo4 to 8, their scores range from 81.42 to 81.52 and all these hypotheses comprise of one HYP, two HBD, and one HBA features. The remaining two models, Hypo9 and Hypo10, have one HYP and three HBA features and show the score of 80.48. These 10 hypotheses contain the same chemical features but differ in terms of their spatial arrangements. Since the ranking score of each hypothesis is quite similar, validation were carried out to select the best model among 10 hypotheses. All 132 HMGR inhibitors in the test set were used for the validation process, and the hit rates for each hypothesis were listed in Table 1. The number of compounds belonging top 10% of the screened hits was used to choose a best pharmacophore model which is able to pick the inhibitors effectively. As a result, Hypo7 was selected as the final pharmacophore model and the pharmacophoric features of the model are shown in Figure 4 along with their inter-feature distance constraints.

**Table 1:** The results of the common feature pharmacophore model generation and validation.

Hypos	Pharmacophore features^a^	Rank	No. of the screened compounds	Top 10%^b^	Active^c^	Moderately active^c^	Inactive^c^
1	HDAA	81.88	120	12	6	-	6
2	HDAA	81.88	120	12	3	-	9
3	HDAA	81.88	118	12	2	-	10
4	HDDA	81.52	105	11	9	1	1
5	HDDA	81.52	107	12	10	2	-
6	HDDA	81.52	105	11	9	2	-
7	HDDA	81.42	106	12	12	-	-
8	HDDA	81.42	105	11	11	-	-
9	HAAA	80.48	122	12	1	-	11
10	HAAA	80.48	120	12	1	-	11

The calculation of hit rates using test set compounds was used for validation.

All the Hypos have Direct hit  = 1111111, Partial hit  = 0000000, and Max. Fit  = 4. Direct or Partial hit means direct or partial match of compound to the pharmocophoric features in each hypothesis. 1, match and 0, no match.

^a^ Pharmacophore features: H, hydrophobic; D, hydrogen bond donor; A, hydrogen bond acceptor.

^b^ The number of compounds which charges ten-percent of the total number of screened compounds.

^c^ Activity scale: Active (IC_50_ <10 nM); Moderately active (10< IC_50_ <100 nM); Inactive (100 nM < IC_50_).

### Virtual screening and drug-like properties filtration

In the drug discovery process, the automated virtual screening is one of the most important procedures to search novel scaffolds. The final pharmacophore model, Hypo7, was used as the 3D query to screen chemical databases including Chembridge (∼50,000), Maybridge (∼60,000), NCI (∼200,000), and Asinex (∼200,000). Initially, 29,045 compounds which have similar chemical groups and spatial properties in Hypo7 were chosen and the screened compounds were tested by calculating their drug-like properties. Among them, 2,590 compounds satisfying Lipinski's rule were selected. To make them more drug-like compounds, ADMET properties were evaluated and then 1, 12, 33, and 35 hit compounds were identified from Chembridge, Maybridge, NCI, and Asinex database, respectively.

### Binding mode analysis of hit compounds using molecular docking and dynamics simulation studies

A total of 81 hit compounds and the two most potent inhibitors (rosuvastatin and atorvastatin) were docked into the active site of HMGR. Rosuvastatin (Inh1) and atorvastatin (Inh2) were assigned as reference compounds to compare their binding modes with hit compounds and to select final hits from the docking studies. Numerous binding poses of each compound were generated and ranked according to GOLD fitness scores. Among the generated poses for the compound, the most populated docking pose with high fitness score was selected and used for further analysis. In the case of Inh1 and Inh2, GOLD fitness scores were 68.94 and 61.83, respectively.

**Figure 5:**
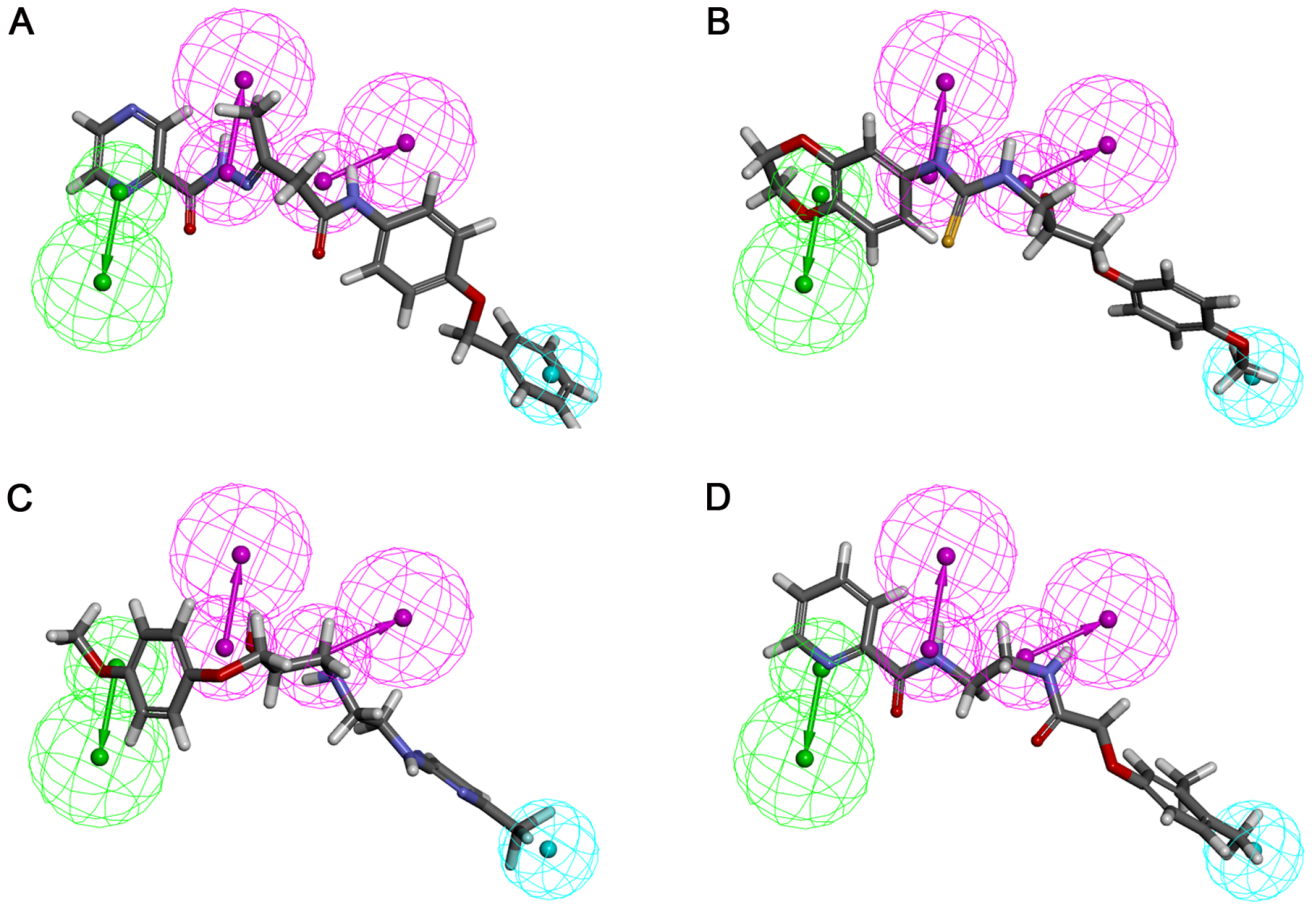
The best pharmacophore model aligned with the retrieved hit compounds obtained from virtual screening. (A) Hit1 (B) Hit2 (C) Hit3 (D) Hit4. HBA, HBD, and HYP features are displayed in green, magenta, and cyan, respectively. Hit compounds are represented in stick model.

**Figure 6:**
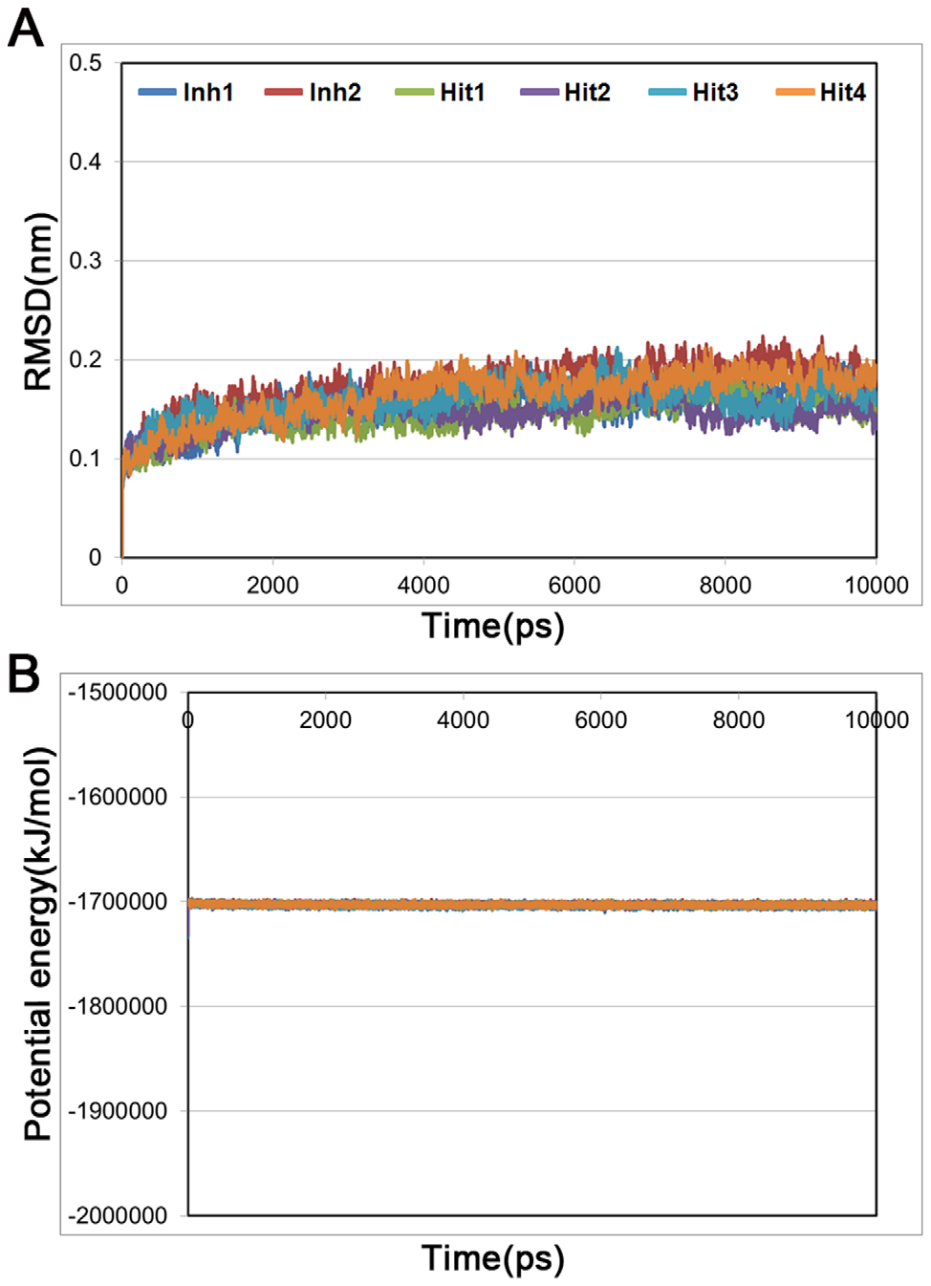
The RMSD and potential energy plots for six complex structures. (A) The RMSD for backbone atoms of the protein. (B) The potential energy of the system. These plots are calculated during 10 ns MD simulations for each complex. Blue, red, green, purple, cyan, and orange lines represent Inh1 (rosuvastatin), Inh2 (atorvastatin), Hit1, Hit2, Hit3, and Hit4, respectively.

**Figure 7:**
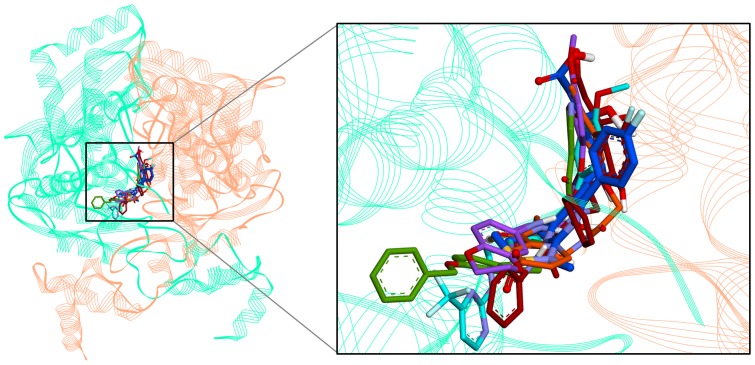
The binding modes of reference and four hit compounds. All compounds in their representative structures were superimposed (left) and enlarged (right). The dimeric structure of HMGR is shown by cartoon model and each monomer is displayed in cyan and salmon color. All compounds are represented as stick model and in the same colors described in Fig. 6. Only polar hydrogen atoms were shown for clarify.

Compounds which have shown a higher fitness score than that of Inh1 and favorable molecular interactions with critical residues were chosen as final hit compounds. The GOLD fitness scores for the selected hit compounds were 79.80, 79.20, 74.18, and 73.30, for hit1, hit2, hit3, and hit4, respectively. Aligning the pharmacophore over the hit compounds revealed that the compounds were well matched with all pharmacophoric features in Hypo7 (Figure 5). The best docked conformation of each compound including reference compounds were used as the initial structure for 10 ns MD simulations to refine their binding conformation. As listed in Table 2, the six systems were subjected to the MD simulation. After finishing the simulation, the root-mean-square deviation (RMSD) of protein backbone atoms and potential energy of the system were calculated to observe the protein stability during the simulation time. As shown in Figure 6A, each system converged well and RMSD values were retained between 0.1 and 0.2 nm until the end of the simulations. The average RMSD values during the simulation time were 0.15 nm, 0.17 nm, 0.14 nm, 0.15 nm, 0.16 nm, and 0.16 nm for Inh1, Inh2, hit1, hit2, hit3, and hit4, respectively. The potential energy also stably maintained in all systems (Figure 6B). These plots indicated that there were no abnormal behaviors in the proteins during the 10 ns MD simulation. The representative structures of six systems were selected from the last 2 ns and used in binding mode analysis. The superimposition of all representative structures showed that the binding patterns of hit compounds were similar to reference compounds (Figure 7).

**Table 2:** The details of six systems used for MD simulations.

No.	System	No. of TIP3P water molecules	No. of Na^+^ counter ions	System size (nm)
1	HMGR + NADPH + Inh1 (Rosuvastatin)	40680	2	7.54×7.85×8.28
2	HMGR + NADPH + Inh2 (Atorvastatin)	40685	2	7.53×7.84×8.29
3	HMGR + NADPH + Hit1	40700	2	8.46×7.77×6.72
4	HMGR + NADPH + Hit2	40697	2	8.46×7.77×6.72
5	HMGR + NADPH + Hit3	40698	2	8.46×7.77×6.72
6	HMGR + NADPH + Hit4	40702	2	8.46×7.77×6.72

**Figure 8:**
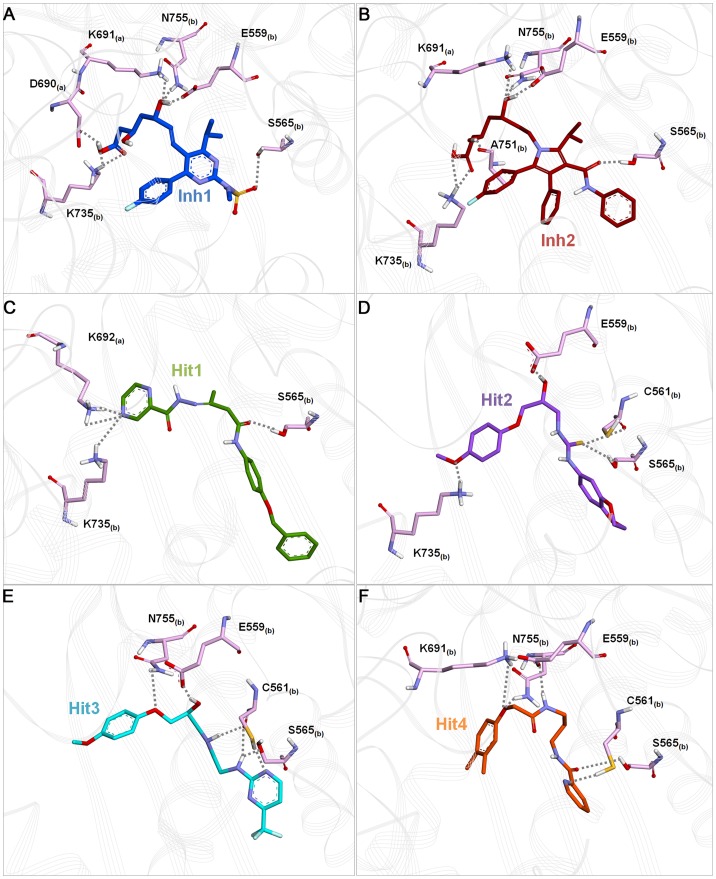
The binding orientations and hydrogen bonding interactions of reference and four hit compounds in the active site of HMGR. (A) Inh1 (B) Inh2 (C) Hit1 (D) Hit2 (E) Hit3 (F) Hit4. All compounds and interacting residues belong to both monomers (called ‘a’ and ‘b’) are represented as pink stick model. Hydrogen bond interactions between compounds and proteins are shown as gray dotted line. Only polar hydrogen atoms were shown for clarify.

The substrate-binding pocket in HMGR was defined by residues Glu559, Ser565, Val683, Ser684, Asp690, Lys691, Lys692, Asn755, Asp767, and His866 [57]. These important residues were also found in the binding of Inh1, Inh2, and 4 hit compounds. In case of reference compounds, Inh1 formed hydrogen bonds with Asp690, Lys691, Glu559, Ser565, Lys735, and Asn755 and Inh2 showed hydrogen bond interactions with Lys691, Glu559, Ser565, Lys735, Ala751, and Asn755 (Figure 8A and 8B). Additionally, both Inh1 and Inh2 were stacked on Arg590 via cation-π interaction. On the other hand, hit1 formed hydrogen bond with Lys692, Ser565, and Lys735 and aminophenol moiety of hit1 was involved in cation-π interaction with Arg568 (Figure 8C). In hit2 binding, hydrogen bonds with Glu559, Cys561, Ser565, and Lys735 were observed (Figure 8D). The benzene moiety and N1 atom of hit2 were participated in cation-π interaction with Arg590 and His752, respectively. Compared to hit2, a similar result was obtained in hit3 binding except for Asn755 and cation-π interactions with Lys691 and Arg568 were identified (Figure 8E). The last compound, hit4 made hydrogen bond interactions with Lys691, Glu559, Cys561, Ser565, and Asn755 while benzene moiety of hit4 formed cation-π interaction with Arg590 (Figure 8F). Taken together, binding mode analysis for hit compounds revealed that they bound to the active site by molecular interactions such as hydrogen bond, hydrophobic, and cation-π interactions and interacting residues are summarized in Table 3.

**Table 3:** Molecular interactions between compound and protein.

	Interacting residues		
Compound	Hydrogen bond (<3.0 Å)	Hydrophobic interaction	Cation-π interaction
**Inh 1**	Asp690, Lys691, Glu559,	Ser684, Asn686, Lys692,	Arg590
	Ser565, Lys735, Asn755	Ala751, Gly560, His752,	
		Ser852, Val853, Ala856,	
		Leu857, His861, Leu862,	
		Val863	
**Inh 2**	Lys691, Glu559, Ser565,	Ser684, Asn686, Asp690,	Arg590, Arg568
	Ala751, Lys735, Asn755	Lys692, Gly560, Arg568,	
		Val853, Leu857, His861,	
		Leu862, Val863, Lys864	
**Hit 1**	Lys692, Ser565, Lys735	Asp690, Lys691, Gly560,	Arg568
		Leu562, Ala751, His752,	
		Asn755, Ser852, Val853,	
		Ala855, Ala856, His861,	
		Leu862, Ser865	
**Hit 2**	Glu559, Cys561, Ser565,	Ser684, Asp690, Lys691,	Arg590, His752
	Lys735	Lys692, Ala751, Asn755,	
		Ser852, Val853, Ala856,	
		Leu857, His861, Leu862,	
		Ser865	
**Hit 3**	Glu559, Cys561, Ser565,	Arg590, Ser684, Cys688,	Lys691, Arg568
	Asn755	Asp690, Leu562, Lys735,	
		Ala751, His752, Ser852,	
		Val853, Ala856, His861,	
		Leu862	
**Hit 4**	Lys691, Cys561, Glu559,	Ser684, Asp690, Lys692,	Arg590
	Ser565, Asn755	Gly560, Leu562, Lys735,	
		Ala751, His752, Val853,	
		Ala856, Leu857, Leu862,	
		Ser865, His866	

The interacting residues forms hydrogen bond, hydrophobic, and cation- π interactions are listed.

**Figure 9:**
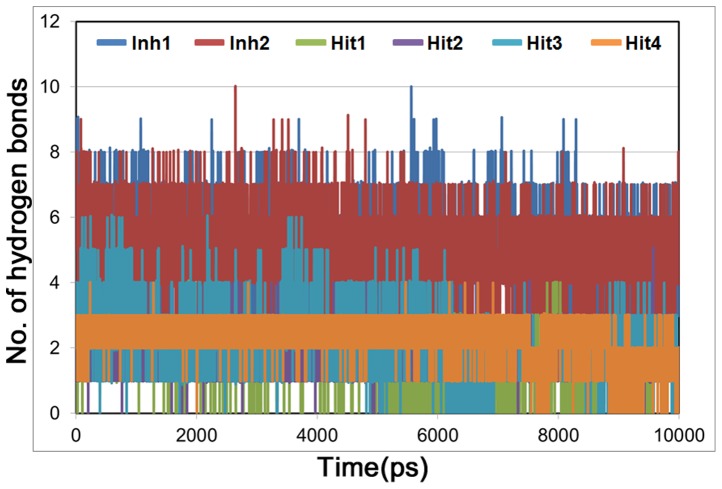
The number of hydrogen bonds between compound and protein during 10 ns MD simulations. Blue, red, green, purple, cyan, and orange colors represent Inh1 (rosuvastatin), Inh2 (atorvastatin), Hit1, Hit2, Hit3, and Hit4, respectively.

**Figure 10:**
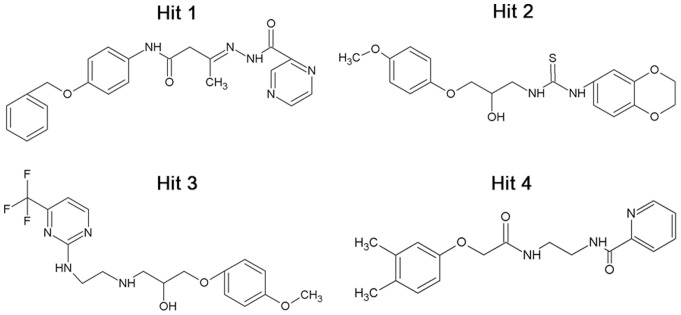
2D structures of final hit compounds. Hit1 and Hit4 were obtained from ASINEX database and the other hits were identified from Maybridge database.

The number of hydrogen bonds between HMGR and each compound were also monitored during the simulation time (Figure 9). Inh1 and Inh2 were maintained almost 5 hydrogen bonds, whereas hit compounds showed less number of bonds than reference compounds. The average numbers of hydrogen bonds between the hit compounds and HMGR were 1.5, 1.9, 2.4, and 2.5 for hit1, hit2, hit3, and hit4, respectively. Although the number of hydrogen bonds in hit compounds was smaller than that of reference compounds, they also formed hydrogen bonds with critical residues and have shown stable interactions in the active site of HMGR. Furthermore, we also calculated binding free energy of hit compounds using their representative structure. The hits have relatively lower binding free energy with −5.22, −5.41, −4.23, and −5.01 kcal/mol for hit1, hit2, hit3, and hit4 compared to the Inh1 (−4.09 kcal/mol) and Inh2 (−2.89 kca/mol). The chemical structures of the final hits and the possible candidates from hit5 to hit16 were shown in Figure 10 and Figure S1. The novelty of the compounds was assessed by the *Pubchem Structure* [58] search tool. These results lead us to the conclusion that the final hit compounds can be suggested as novel scaffolds to design potent inhibitors of HMGR.

## Conclusions

Hypercholesterolemia is known as a significant factor for the coronary artery disease that is a leading cause of death in the world. For the treatment of this disease, inhibition of HMGR enzyme is considered as the most general way. Statins were already well known as HMGR inhibitors and commonly used in anti-hypercholesterolemia drugs. But several studies indicated that statins have some side effects such as distal muscle weakness, headache, and sleep disorders. So there is continuous need for developing novel HMGR inhibitors with fewer side effects. In the present study, we developed common feature pharmacophore models with the training set compounds. The generated models were validated using the test set compounds and then Hypo7 was selected as the final model. In the subsequent virtual screening using Hypo7, 29,045 new compounds were retrieved from chemical databases namely Chembridge, Maybridge, NCI, and Asinex. The screened compounds were filtered by applying Lipinski's rule of five and ADMET properties. A total of 81 compounds which satisfied all the drug-like properties were used in molecular docking study to obtain reasonable binding conformations in the active site of HMGR. Based on the GOLD fitness scores and molecular interactions with active site residues, 4 compounds shown higher fitness scores than that of reference compounds were selected as final hit compounds and the hits were subjected to 10 ns MD simulations to refine their binding modes. After 10 ns MD simulations, the representative structures of the reference and hit compounds were compared. The binding mode analysis revealed that our final hits bound to HMGR in a similar manner as reference compounds and formed hydrogen bonds with critical residues like Lys691, Lys692, Glu559, Ser565, Lys735, and Asn755. Also, hydrophobic and cation-π interactions between hit compound and the active site residues were observed. Even though the final hits have less number of hydrogen bonds than that of reference compounds, their interactions with essential residues were appreciable and they also have shown favorable binding free energies. From the above discussion, we can propose the final hit compounds as virtual candidates for HMGR inhibitors. Further, we believe that new scaffolds including final hits obtained from our results will be helpful in the designing of novel anti-hypercholesterolemia drugs.

## Supporting Information

Figure S1 **2D structures of the possible candidate scaffolds.** The 12 top-ranking compounds, from hit5 to hit16 were also shown reasonable interactions in the active site of HMGR but not sufficient to be final hits.
(TIFF)
