# Supplementary figures and images for: Exploration of Virtual Candidates for Human HMG-CoA Reductase Inhibitors Using Pharmacophore Modeling and Molecular Dynamics Simulations
Source: PLoS One. 2013 Dec 30;8(12):e83496. doi: 10.1371/journal.pone.0083496 (PMC3875450; doi:10.1371/journal.pone.0083496)

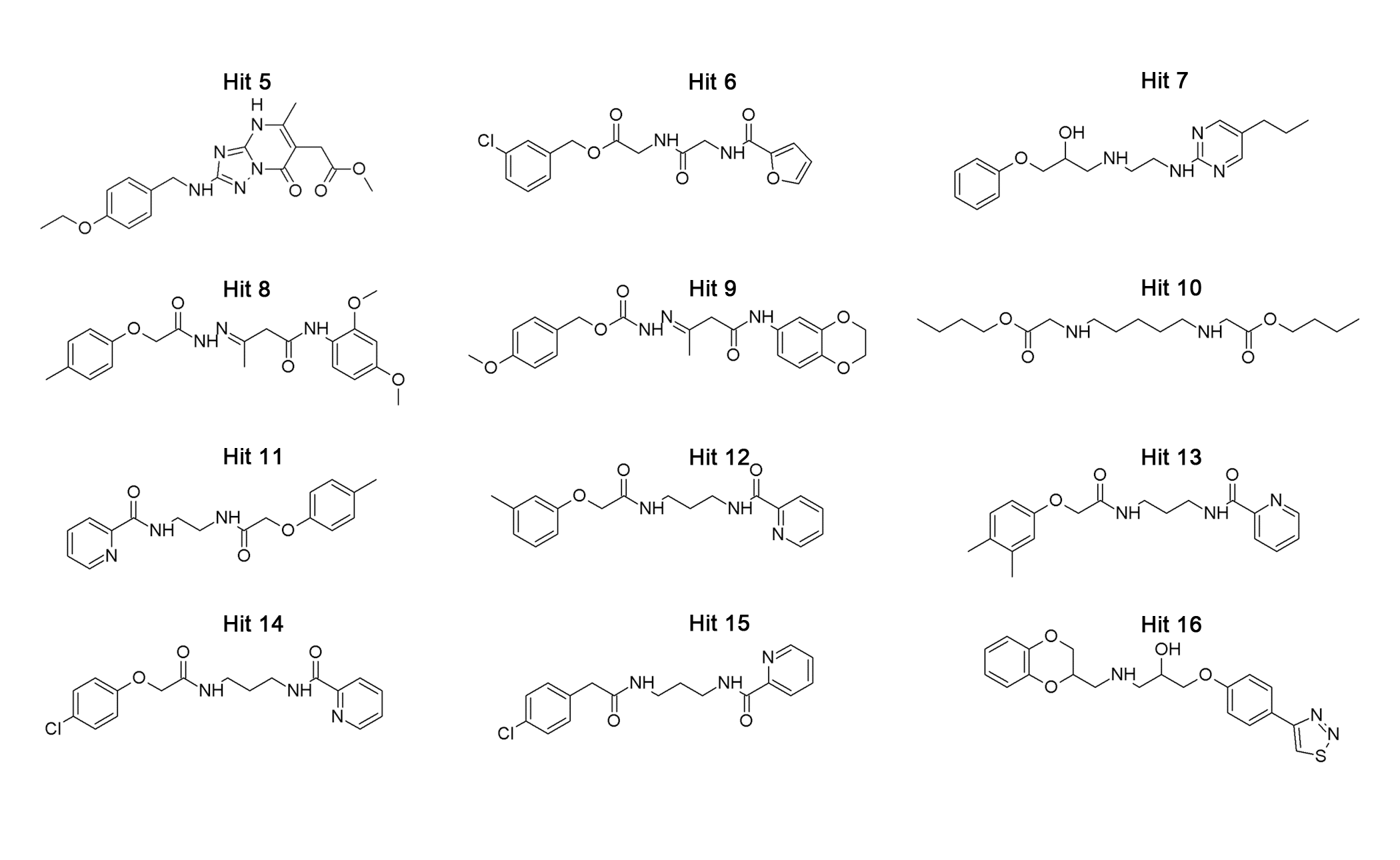

Supplement: Figure S1 — 2D structures of the possible candidate scaffolds. The 12 top-ranking compounds, from hit5 to hit16 were also shown reasonable interactions in the active site of HMGR but not sufficient to be final hits. (TIFF) [file pone.0083496.s001.tiff]
